# TENS Improves Cisplatin-Induced Neuropathy in Lung Cancer Patients

**DOI:** 10.3390/medicina58101405

**Published:** 2022-10-06

**Authors:** Sanja Tomanovic Vujadinovic, Nela Ilic, Ivan Selakovic, Una Nedeljkovic, Nevena Krstic, Natasa Mujovic, Emilija Dubljanin Raspopovic, Dragana Jovanovic

**Affiliations:** 1 Faculty of Medicine, University of Belgrade, 11000 Belgrade, Serbia; 2 Center for Physical Medicine and Rehabilitation, University Clinical Center of Serbia, 11000 Belgrade, Serbia; 3 Clinic of Pulmonology, University Clinical Center of Serbia, 11000 Belgrade, Serbia

**Keywords:** lung cancer, cisplatin, cisplatin-induced neuropathy, neuropathy, neuropathic pain, TENS, quality of life

## Abstract

*Background:* Cisplatin-induced peripheral neuropathy is a common complication of cisplatin therapy, which develops in most patients with lung cancer. There are no effective preventive measures and once it occurs there is no effective therapy, except symptomatic. In this study, we aimed to assess the effect of transcutaneous electrical nerve stimulation (TENS) therapy on the pain intensity and the quality of life of patients with cisplatin-induced neuropathy. *Material and Methods:* A prospective cohort study was performed from 2013 to 2018, at the Clinical Center of Serbia. After the initial evaluation of 106 newly diagnosed patients with lung cancer, 68 patients did not have peripheral neuropathy. These 68 patients continued in the study and started the cisplatin chemotherapy. Forty of these patients developed cisplatin-induced neuropathy, which was manifested by neuropathic symptoms and proven by ENG examination. All patients with cisplatin-induced neuropathy were treated with TENS therapy. Their neuropathic pain and quality of life were evaluated using the following questionnaires at diagnosis, after cisplatin therapy and after four weeks of TENS use: DN4, VAS scale, EORTC QLQ-C30 and FACT-L. *Results:* Two thirds (68%) of the patients with cisplatin-induced neuropathy were male and the majority were smokers (70%). Adenocarcinoma was the most common (38%), followed by squamous (33%) and small-cell carcinoma (28%). The application of TENS therapy had a positive effect on reducing the neuropathic pain and increasing the quality of life for patients with painful cisplatin-induced neuropathy. The VAS and DN4 scores significantly decreased after TENS therapy, in comparison to its values after cisplatin therapy (*p* < 0.001). After TENS therapy, patients had significantly higher values in most of the domains of EORTC QLQ-C30 and FACT- L, in comparison with the values after cisplatin therapy (*p* < 0.001). *Conclusion:* The application of TENS therapy has a positive effect on reducing neuropathic pain and increasing the quality of life for patients with lung cancer and cisplatin-induced neuropathy.

## 1. Introduction

Lung cancer is the second leading malignant disease in the world. It is estimated that approximately 2.2 million new cases of lung cancer are diagnosed annually. Lung cancer is the most common cause of death from malignant tumors in men, and, after breast and colorectal cancer, it is the leading cause of death from malignant tumors among women [1]. Neurological complications from lung cancer are frequent. A recent study showed that 64% of patients with lung cancer show signs of neurological deficits [2]. Early diagnosis and evaluation are important for the preservation of neurological functionality, but also for the choice of therapy, which can worsen the symptoms of neuropathy and affect its further prognosis [3,4].

Cisplatin-induced peripheral neuropathy is a common complication of cisplatin therapy, which develops in most patients. There are no effective preventive measures and once it occurs there is no effective therapy, except symptomatic. Cisplatin causes axonal neuropathy that predominantly affects large myelinated sensory fibers. The primary site of damage is the dorsal root ganglia, but peripheral nerves can also be affected. Unlike the central nervous system, the peripheral nervous system does not have a blood–brain barrier, and, therefore, allows contact with chemotherapeutics and other neurotoxins. The lack of the lymphatic system and cerebrospinal fluid surrounding the peripheral nerves allows harmful substances to build up around the nerve tissue, worsening the neuropathic damage [5]. Cisplatin-induced peripheral neuropathy is dependent on the total cumulative dose and usually develops after a dose above 300 mg/m^2^. At a cumulative dose of 500 to 600 mg/m^2^, almost all patients have objective evidence of neuropathy. However, a significant inter-individual variation persists as a consequence of the genetic polymorphisms of the enzymes related to cisplatin metabolism (e.g., glutathione S-transferase). A greater intensity of the cisplatin dose per unit of time does not affect the severity of neuropathy [6,7,8].

When a mild neuropathy occurs, the continuation of therapy at the full dose is recommended. In the case of more severe neuropathy, a change in the therapy is considered, e.g., replacement of cisplatin with carboplatin. Even when cisplatin is stopped, in 30% of patients the symptoms of neuropathy worsen over the following months. Patients usually experience coldness, tingling, itching, burning, numbness and pain. Moreover, symptoms can be so intense that they are perceived as electric shocks or burns, which worsen when a part of the patient’s body in which neuropathic symptoms are present is touched [3,9,10]. The risk factors for the persistence of neuropathy are age, smoking history, alcohol use, arterial hypertension and some hereditary factors [11,12,13].

Some patients improve over time, although only partially [14,15]. The guidelines of the European Federation of Neurological Societies (EFNS) state that there is no clear and effective therapeutic option, nor a targeted group of drugs that are characterized as the most effective in the treatment of neuropathic pain [16]. The therapeutic approaches for the majority of neuropathy cases are the use of antidepressants, anticonvulsants, opioids and different modalities of physical therapy, such as transcutaneous electrical nerve stimulation (TENS) [16,17,18,19]. TENS therapy is applied as the mediated neural stimulation causes the release of opioids that suppress pain. A recent systematic review concluded there is not enough evidence to recommend TENS therapy as a standard procedure for the treatment of chemotherapy-induced peripheral neuropathy and that well-designed trials with adequate follow-ups are needed to define the specific protocols for its effective use. However, authors have stated that TENS therapy is safe and an easy-to-use procedure that may be used in an attempt to relieve the pain symptoms in cancer patient population [20]. Therefore, the aim of this study was to examine the effect of TENS therapy on the pain intensity and the quality of life in a cohort of newly diagnosed lung cancer patients, with cisplatin-induced neuropathy.

## 2. Materials and Methods

The study was conducted from December 2013 to January 2018 at the Clinic of Pulmonology, the Clinic of Physical Medicine and Rehabilitation and the Clinic of Neurology at the University Clinical Center of Serbia. The research protocol was approved by the Ethics Committee of the Clinical Center of Serbia, and written informed consent was obtained from each patient. The inclusion criteria were as follows: newly diagnosed histopathologically confirmed lung cancer, ECOG performance status ≤ 3 and patients over 18 years of age. Patients with any intoxication (ethylic etiology or elevated blood sugar levels), diabetes, increased levels of nitrogen substances in renal failure, increased levels of thyroid hormone in hyperthyroidism, associated systemic diseases, sarcoidosis, hematological diseases, hepatitis, HIV, previous radiotherapy, trauma or surgical intervention (up to 6 months), vitamin B12 deficiency and gammopathy were excluded from the study.

**Figure 1:**
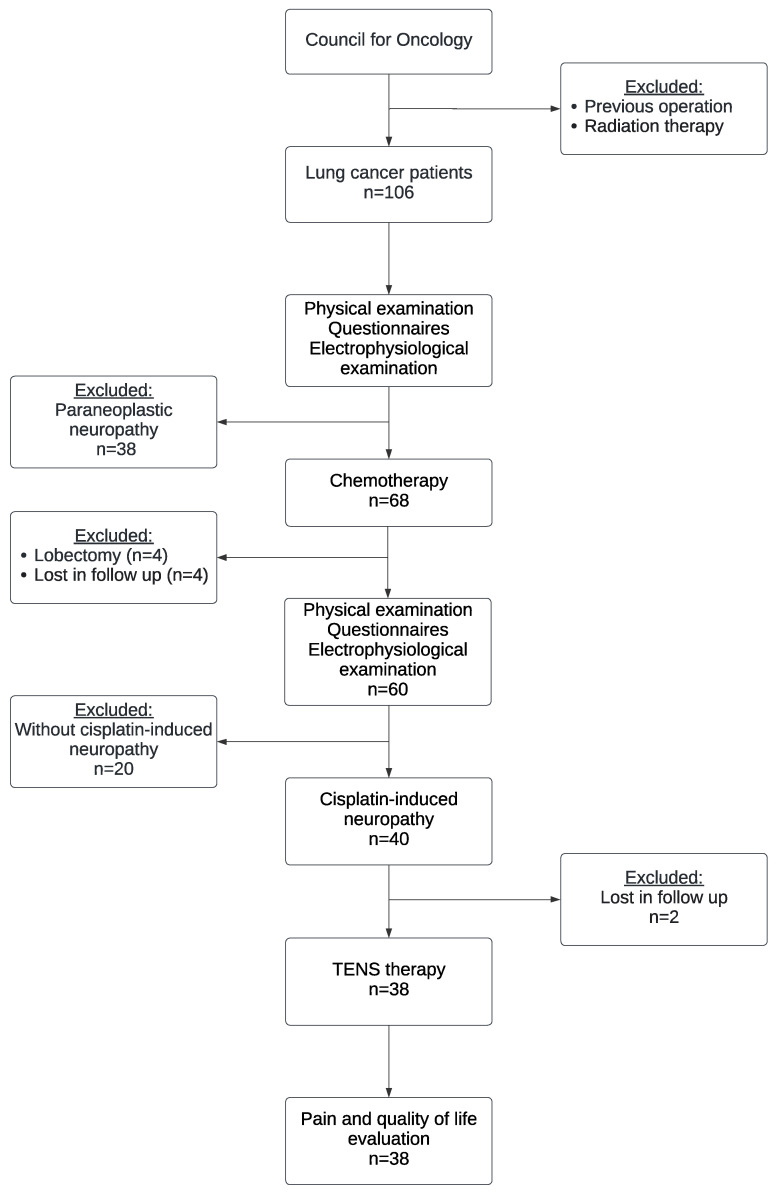
Flow chart.

The initial evaluation of 106 newly diagnosed patients with lung cancer showed that 68 patients did not have peripheral neuropathy (Figure 1). They continued in the study and started cisplatin chemotherapy. After the fourth cycle of chemotherapy, the patients were evaluated again for presence of peripheral neuropathy (cisplatin-induced) and 8 patients in total were excluded: 4 due to lobectomy and 4 were lost in follow-up. Forty patients developed cisplatin-induced neuropathy, manifested by neuropathic symptoms and proven by ENG examination. Twenty patients who did not develop cisplatin-induced neuropathy after 4 months of chemotherapy were excluded from the study. All patients with cisplatin-induced neuropathy were treated with TENS therapy. Neuropathic pain and quality of life were evaluated before and after 4 weeks of TENS use. Patients were asked to specify the localization of pain and the painful region was marked on the diagram in the patients’ medical records.

The following tests and questionnaires were used in the study:

*Sociodemographic questionnaire.* The following sociodemographic characteristics were assessed: gender, age, smoking habits (current smokers, ex-smokers, non-smokers, years of smoking, and number of cigarettes smoked per day), cancer stage and histopathology.

*Neurological symptom survey.* Neurological symptom survey consisted of 26 questions assessing the presence of unilateral or bilateral sensory and/or motor neurological symptoms on the upper and lower extremities. Possible answers were “yes” or “no”, and the presence of bilateral neurological symptoms suggested the diagnosis of neuropathy.

*Medical Research Council (MRC) sum score.* MRC sum score is a quantitative muscle strength assessment tool used to assess the strength of the muscles of the upper and lower extremities. The assessment of the gross muscle strength of the muscles is conducted bilaterally, whereby the ranking is from 0 (absence of movement) to 5 (normal muscle strength). The sum score ranges from 0 (complete loss of all 4 limbs) to 80 (full strength of all tested muscles) [21].

*Douler Neuropathique in 4 questions (DN4).* DN4 is a survey questionnaire that consists of two parts and is used in the diagnosis of neuropathic pain. In the first part, patients were interviewed using two questions, with 7 items that describe the quality of pain and sensitive symptoms felt by the patient. The second part was based on a clinical examination and had two questions with 3 items that examined a reduced sensation to touch or needle prick (hypesthesia) and whether pain increased or appeared when the brush was lightly pressed over the skin (allodynia). All affirmative answers were scored with one point, and all negative answers were scored with 0 points. The results were added up and a score ≥ 4 indicated pain of neuropathic origin [22].

*Electrophysiological examination (ENG).* Peripheral nerve conduction studies, both conventional and standardized, were performed bilaterally for the upper (median and ulnar nerves) and lower extremities (tibial and sural nerves), using surface stimulation electrodes, and with the registration of evoked responses using the Sinergy EMG device (Viasis, UK). The filter settings were 3 Hz–10 kHz for motor conduction studies and 20 Hz–2 kHz for sensory conduction studies. The filters for the F-wave were set between 30 Hz–10 kHz, with the application of the built-in notch filter (50/60 Hz) function in the EMG machine. During stimulation and monitoring, the following electrophysiological indicators were recorded and then analyzed: compound muscle action potentials (CMAPs) in the m. abductor pollicis brevis, m. abductor digiti minimi, m. extensor diggitorum brevis and m. flexor hallucis brevis. Furthermore, distal and proximal muscle latencies, motor nerve conduction velocity (MNCV) and late latencies response (LLR) were recorded for the same CMAPs. Sensory nerve action potentials (SNAPs) were examined with antidromic stimulation for the median, ulnar and sural nerves bilaterally, using ring electrodes on the 2nd and 5th fingers for the median and ulnar nerves, as well as on the big toe for the sural nerve. MNCVs were registered between the wrist and the cubital fossa (for the median nerve); the wrist and the elbow (for the ulnar nerve); the ankle joint (region of the medial malleolus) and the popliteal fossa (for the tibial nerve); and between the ankle joint and the proximal position below the head of the fibular bone (for the peroneal nerve). The sensory nerve conduction velocities were calculated for the distal segments of the extremity using the antidromic method. Amplitudes of the CMAPs and SNAPs were measured from the initial deflection (deviation from the isoelectric line) to the first negative peak. Lower reference limits were defined as mean values, reduced by two standard deviations in relation to the normative data of the laboratory, registered in the same way. For each nerve, we defined typical electrophysiological patterns of abnormalities, as follows: demyelinating pattern, axonal pattern, and combined axonal/demyelinating damage pattern. The presence of abnormalities in at least two peripheral nerves was considered as electrophysiological impairment.

*Visual Analogue Scale (VAS).* VAS scale is an instrument used to measure the subjective feeling of pain intensity. It is designed as a horizontal line, containing indicators for the absence of pain, through medium intensity to the maximum sensation of pain. The patient marks their perception of pain at that moment, at a certain point on the scale. The numerical value of pain corresponds to the patient’s subjective feeling of pain [23].

*Questionnaire on assessment of quality of life of patients suffering from malignant disease (EORTC QLQ-C30).* EORTC QLQ-C30 is a questionnaire used to assess the quality of life in patients with a malignant disease [24]. The questionnaire consists of 30 questions related to the quality of performing daily activities, in relation to physical condition and general health condition of the patient. It includes the condition of organic systems, presence of depression, subjective feeling of the patient in relation to the disease and family, as well as self-assessment of the general health condition in the period of 7 days before testing. The EORTC QLQ-C30 score ranges from 0 to 100, and a higher score indicates a better quality of life. The questionnaire contains five multi-item functional domains. This includes physical functioning (PF), role functioning (RF), emotional functioning (EF), cognitive functioning (CF) and social functioning (SF). A higher score indicates better functioning. Moreover, nine symptom domains were assessed, where multiple symptoms indicated poor functioning. Symptoms’ scale domains are as following: fatigue (FA), nausea and vomiting (NV), pain (PA), shortness of breath (DY), insomnia (SL), loss of appetite (AP), constipation (CO), diarrhea (DI) and financial difficulties (FI). Additionally, two independent domains, global health status (QL) and QLQ total score (QLQ Total) were assessed.

*Questionnaire for assessing the quality of life of patients undergoing lung cancer therapy (FACT-L).* FACT-L is used for the assessment of the quality of life in patients undergoing therapy for lung cancer [25]. The questionnaire consisted of 5 domains describing the patient’s physical condition, social and family environment, emotional condition, functional condition, and additional concerns in the last 7 days before chemotherapy. Physical state (PWB); social/family state (SWB); emotional state (EWB); functional state (FWB); FACT-G final/total score (PWB + SWB + EWB + FWB); lung carcinoma (LCS); FACT-L end/total score (PWB + SWB + EWB + FWB + LCS); and FACT-L-TOI-Study Outcome Index (PWB + FWB + LCS) represented FACT-L domains. Answers were scored from 0 to 4, where a higher score indicated a better quality of life. Permission to use FACT and EORTC questionnaires was granted.

*TENS use.* All patients with cisplatin-induced neuropathy were treated with TENS for 4 weeks, 5 days a week and each session lasted 30 min. No patient underwent TENS more than once a day during the study. The characteristics of electrical currents used were as follows: 2 channels, 4 outputs, pulse frequency 80 Hz/s, pulse duration 200 µs, and electric current strength approximately 60 mA. The electric current strength was adjusted according to the patients’ subjective feelings, until the feeling of tingling without pain or discomfort was felt. TENS electrodes were positioned diagonally, with the center in the zone of pain, so that electric current passed through the target zone. The patients felt a pleasant tingling sensation that masked the sensation of pain. During the study period, patients did not receive gabapentinoids or any analgesic drug therapy for neuropathic pain.

*Statistical analysis.* Baseline characteristics and sociodemographic data were stratified by the presence of neuropathy in patients, as well as the diagnosis of neuropathy before and after cisplatin therapy. Numerical data were presented as mean, with 95% confidence interval, or with minimum and maximum value. Categorical variables were summarized as absolute numbers with percentages. Changes in examined variables from baseline (time at diagnosis) to after cisplatin and after TENS therapy were evaluated by repeated measures ANOVA. Interactive line graphs for presenting changes during time in examined variables were created using an interactive graph tool [26]. In all analyses, the significance level was set at 0.05. Statistical analysis was performed using IBM SPSS statistical software [27].

## 3. Results

A total of 106 patients with newly diagnosed lung cancer were initially included in the study. The majority were male, with a mean age of 64 (47–83) years. Most patients were diagnosed with the third stage of the disease (53%), a squamous type of lung cancer (37%) and a non-small-cell carcinoma (70%). The characteristics of the patients without and with neuropathy on initial evaluation are shown in Table 1.

Out of 106 patients with lung cancer, 38 had neuropathy at the time of diagnosis and were excluded from further evaluation. After four months, 40 patients developed cisplatin-induced neuropathy. Two thirds of the patients with cisplatin-induced neuropathy were male (68%). The youngest patient was 47 years old, while the oldest was 82 years old. The majority of the patients with lung cancer and cisplatin-induced neuropathy were smokers (70%). Adenocarcinoma was the most common type of lung cancer in patents with cisplatin-induced neuropathy (38%), followed by squamous (33%) and small-cell carcinoma (28%) (Table 1).

All patients with cisplatin-induced neuropathy underwent TENS therapy for their neuropathic pain, and their quality of life, using different questionnaires, was measured at diagnosis, after cisplatin therapy and after TENS therapy.

**Figure 2:**
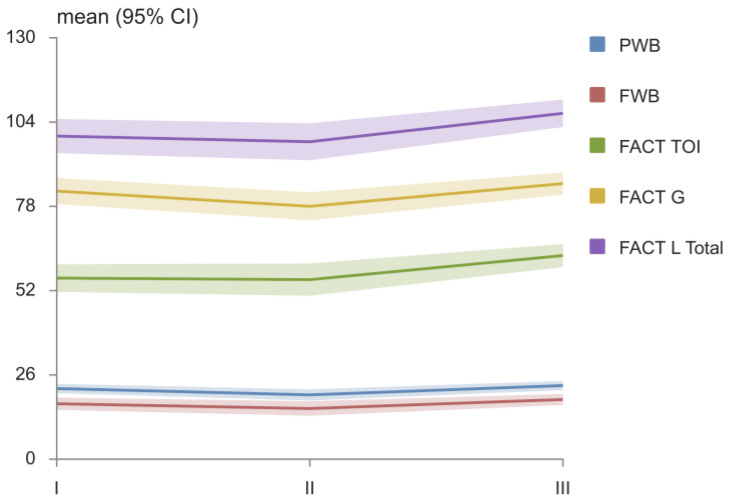
FACT L—functional assessment of cancer therapy–lung; PWB—physical wellbeing; FWB—functional wellbeing; FACT L TOI-FACT-L trial outcome index (TOI); FACTG total score–PWB + SWB + EWB + FWB; and FACT L TOTAL score–PWB + SWB + EWB + FWB + LCS.

In Table 2, the domains of the FACT questionnaire are presented. After the application of TENS therapy, patients had significantly higher values of PWB, FWB, LCS, FACT L TOI, FACT G, and FACT L TOTAL, in comparison with the values after cisplatin therapy. Additionally, at diagnosis, the values of EWB and LCS were lower, whereas the values of FACT L TOI and FACT L TOTAL were higher than they were after TENS therapy. The values of SWB were significantly higher and the values of LCS were significantly lower at diagnosis than they were after cisplatin therapy (Table 2). The changes in the PWB, FWB FACT L TOI, FACT G, and FACT L TOTAL over time are shown in Figure 2.

**Figure 3:**
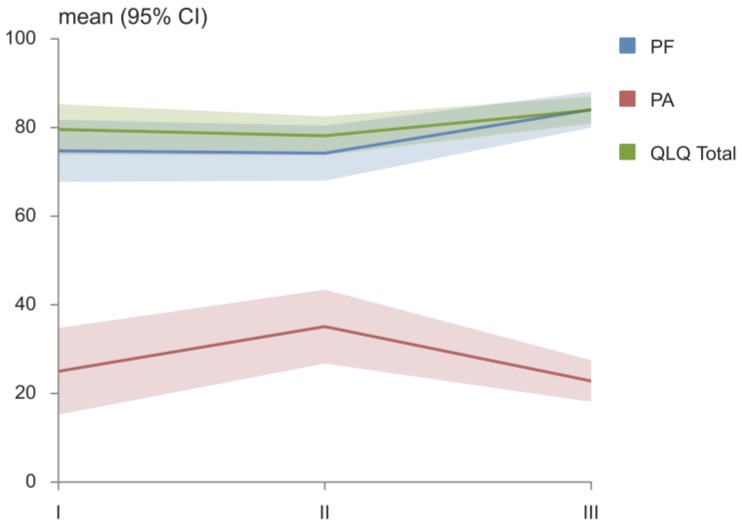
EORTC QLQ–C30—European Organization for Research and Treatment of Cancer–Quality of life questionnaire C30; PF—physical functioning; PA—pain; QLQ Total—QLQ total score.

The EORTC QLQ-C30 questionnaire and its domains were evaluated at the time of diagnosis, after cisplatin therapy and after TENS therapy, and the results are presented in Table 3. After the TENS therapy, the average values of the PF and FI domains were significantly higher than they were at diagnosis. After 4 months of cisplatin therapy, the values of PF, RF, QL and OLQ Total were significantly lower and the values of PA, SL, AP and DI were significantly higher, in comparison to the values of these domains after TENS therapy. The values of the EF, CF, SF, FA, NV, DY and CO domains of the EORTC QLQ-C30 questionnaire remained similar during the follow up (Table 3). The changes in the PF, PA and QLQ Total during the follow up are shown in Figure 3.

**Figure 4:**
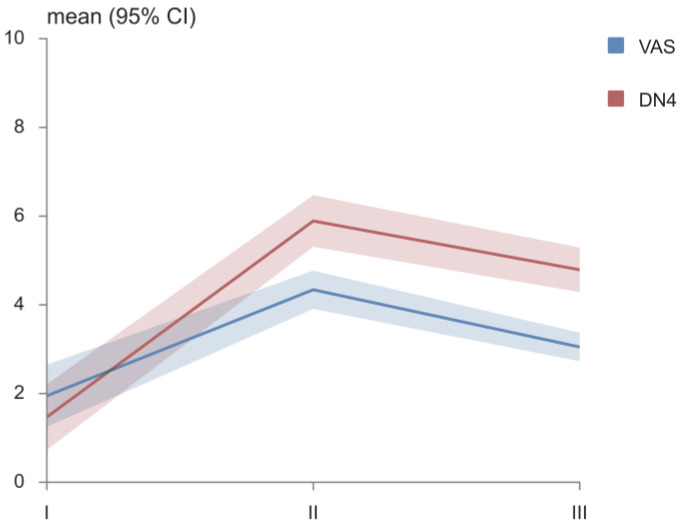
VAS and DN4. VAS—Visual Analogue Scale; DN4—Douler Neuropathique in 4 questions.

The value of the VAS score changed during the follow up (Figure 4). The VAS score at diagnosis was significantly lower than it was after cisplatin therapy (*p* < 0.001). In comparison to the VAS score after cisplatin therapy, the value of the score significantly decreased after TENS therapy (*p* < 0.001).

The neuropathic pain in the patients, measured using DN4 questionnaire, was at its highest level after cisplatin therapy (Figure 4). After applying TENS therapy, the neuropathic pain was reduced (*p* < 0.001).

## 4. Discussion

We performed a prospective cohort study to assess the effects of TENS therapy on patients with cisplatin-induced neuropathy and concluded that the application of TENS therapy has a positive effect on reducing the neuropathic pain and increasing the quality of life for patients with painful cisplatin-induced neuropathy.

The etiology of neuropathies in malignant patients is considered to be multifactorial, including the metabolic and nutritional deficits, but also including unrecognized factors, which typically appear in the advanced stage of the underlying disease [28,29]. The causal connection of neuropathies in the older patient population is particularly controversial, since numerous possible causes of peripheral nerve damage are already present [30]. McLeod reported that, depending on the diagnostic criteria, up to 50% of cancer patients develop peripheral neuropathy [31]. The mechanism of damage is associated with onconeural antibodies and onconeural antigen-specific T-lymphocytes. However, the absence of known onconeural antibodies in patients with typical clinical manifestations of neurological damage does not rule out the possibility of paraneoplastic neuropathy. These forms of neuropathies often foresee the appearance of the underlying disease, and, sometimes, independently of cancer, create a high level of functional disability (e.g., subacute sensory neuronopathy) [32,33].

Patients with lung cancer often develop chemotherapy side effects that can impair further therapy implementation. Previously published studies reported that 30% of peripheral neuropathies are due to the neurotoxicity of oncological drugs [3]. Symptoms such as tingling, burning, prickling, pain, cold feet and hands, itching, loss of proprioception, weakness, and gait disturbance can be unbearable for patients and can lead to the termination of therapy. Neurotoxicity is attributed to the cumulative drug dose, treatment intensity and pharmacokinetics [14,34,35].

Differentiating acquired sensory neuropathies from other neuropathies is important for the selection of adequate therapy. The systematic literature review published by Ruelle et al. in 2017, included case reports (*n* = 31), case series (*n* = 30) and 10 retrospective studies. One hundred and forty-two cases of lung cancer were associated with neurological symptoms and diagnosed as sensory neuropathy [36]. The study showed a male predominance among patients with neuropathy (68%), with a mean age of 63 years. The most frequently described histopathological type of lung cancer was small-cell lung cancer (89.5%). Due to the strict exclusion criteria, our study did not include patients with associated paraneoplastic syndromes, however, in the study by Ruelle et al., subacute sensory neuropathy was associated with other paraneoplastic neurological syndromes in 68 patients, as follows: autonomic neuropathy (23%), paraneoplastic cerebellar degeneration (9%), paraneoplastic limbic encephalitis (7%) and Lambert Eaton myasthenic syndrome (6%) [36]. In our study, the patients were strictly selected, excluding all patients with possible causes of neuropathy, such as diabetes mellitus, alcoholism, gammopathy, heavy metal load, cachexia and vitamin deficiency. After four cycles of cisplatin chemotherapy, neuropathy was diagnosed in 67% of the patients. Data from the literature show that cisplatin-induced peripheral neuropathy develops after a cumulative dose of cisplatin over 300 mg/m^2^ and that, with a cumulative dose of 500 to 600 mg/m^2^, almost all of the patients had objective evidence of neuropathy [6,7,8,37]. In our research, electrophysiologically proven neuropathy was manifested at a dose of 400 mg/m^2^ cisplatin.

Although a large number of cancer patients develop chemotherapy-induced neuropathy, there is still insufficient data in the literature regarding the use of TENS for the treatment of neuropathic pain in this patient population. Studies have been conducted in patients with diabetic polyneuropathy, peripheral mononeuropathy of traumatic origin, in painful cervical radiculopathy, and in patients with chronic pain that includes a neuropathic component [38,39,40,41,42,43,44,45,46]. Based on nine controlled studies, with 200 treated cases of neuropathic pain, EFNS states that TENS therapy is superior to a placebo in reducing pain [18]. In a study of 40 patients with diabetes, the effect of TENS therapy on central and peripheral neuropathic pain was evaluated and compared [19]. Pain intensity, pain quality and functional capacity were assessed with the VAS scale, the Neuropathic Pain Scale, and the Brief Pain Inventory. As in our study, 20 sessions of 30 min each were applied for 4 weeks. The pain parameters in both groups were significantly reduced (*p* < 0.05). The peripheral neuropathic pain group (PNP) showed more complete improvements than the central neuropathic pain group (CNP). The mean pain intensity in the PNP group was reduced by 38%, and in the CNP group by 15%. Studies have reported that improvements in terms of pain reduction are significant when the improvement is greater than 30% [17]. The results by Dubinski et al., published in 2010, showed that the application of TENS therapy was superior in reducing neuropathic pain, in comparison to high-frequency muscle stimulation [47]. An increasing number of studies include indicators of quality of life, in addition to the basic characteristics and indicators of disease progression [48,49,50].

A study by Siemens, published in 2020, evaluated the efficacy and safety of TENS therapy as an additional therapy for cancer patients. To assess the patients’ quality of life, the EORTC QLQ C30 questionnaire was used in a placebo and intervention group, as well as the DN4 for the assessment of neuropathic pain. Their results imply that TENS therapy is a safe treatment method but the difference in the analgesic effect between the intervention and the placebo group was not significant [48]. According to our results, the level of neuropathic pain measured using the DN4 decreased from 5.9 (the mean value after cisplatin therapy) to 4.8 (the mean value after TENS therapy), which was a significant decrease (*p* < 0.001). The effectiveness of TENS therapy was also confirmed in our study by measuring the VAS score. The value of the VAS significantly decreased, from a mean value of 4.3, to one of 3.0 after TENS therapy. Additionally, most of the domains of the EORTC QLQ C30 questionnaire showed significant improvement in the quality of life for our study group after TENS therapy.

Fiorelli et al. reported that TENS treatment created a greater reduction in the postoperative pain intensity of lung cancer patients who had undergone a thoracotomy, than it had in the placebo group [51]. Fereira et al., in 2011, also assessed the level of pain in lung cancer patients. TENS therapy was applied to 30 patients, who were randomly divided into placebo and control groups two days after they had undergone a thoracotomy. The VAS was measured before the TENS therapy, immediately after and one hour later. The authors of the study concluded that TENS therapy had an analgesic effect immediately after application. One hour after TENS use, the reduction in pain was not observed [52]. TENS therapy for post-thoracotomy pain was maintained for 30 min, in the study of Solak et al. [53], 45 min in the study of Chandra [54] and for 48 h, continuously, in the study of Erdogan et al. [55]. This variability in treatment duration may explain the differences in the results. Most of the aforementioned authors agreed that, in general, TENS therapy could be associated with a reduction in the pain of patients who had undergone a thoracotomy, but that the duration of its effect, the appropriate treatment duration and the treatment parameters still need to be further examined and clarified.

In a study by De Santana et al., high and low frequency TENS therapy was used for a reduction in postoperative pain. One group was treated with 100 Hz (high), the second with 4 Hz (low) frequency, and the third group with a placebo. TENS therapy was applied for 20 min, at a pulse duration of 100 ms. Electrodes were placed around the incision. The pain intensity was measured by the numeric rating scale. The pain was significantly reduced, in comparison to the placebo group, immediately after the TENS treatment [56]. To attain pain relief in our study, TENS therapy with a pulse frequency of 80 Hz/s, a pulse duration of 200 µs and an electric current strength of 60 mA was applied for 30 min.

The mixed results of TENS therapy’s effectiveness for reducing neuropathic pain presented in the literature can be explained by the differently applied duration of the TENS therapy, as well as by the variations in the TENS pulse widths, rates, frequencies and electrode site positions. Establishing field-wide methodological and analytical standards may allow researchers to gain additional insights into the role of TENS therapy in reducing pain for patients with lung cancer and cisplatin-induced neuropathy.

This study has some limitations. These include the small sample size, having patients recruited from a single center and the absence of a control group. In addition, the fact that we did not monitor the long-term effects (beyond more than 4 weeks) of TENS therapy in reducing the patients’ neuropathic pain can be considered as another limitation of the study and an opportunity for future research. Further studies investigating the use of TENS therapy in patients with chemotherapy-induced neuropathy are needed in order to confirm the results obtained in our study.

## 5. Conclusions

The findings of this study support the role of TENS therapy in reducing neuropathic pain and in improving the quality of life for patients with lung cancer and cisplatin-induced neuropathy.

## Figures and Tables

**Table 1:** Characteristics of study population.

	All Lung Cancer Patients *n* = 106	without Neuropathy *n* = 68	with Neuropathy *n* = 38	Patients without Neuropathy before Cisplatin Therapy *n* = 60	Patients without Neuropathy after Cisplatin Therapy *n* = 20	Cisplatin-Induced Neuropathy *n* = 40
Gender, *n* (%)						
Male	74 (70%)	45 (66%)	29 (76%)	42 (70%)	15 (75%)	27 (68%)
Female	32 (30%)	23 (34%)	9 (24%)	18 (30%)	5 (25%)	13 (32%)
Age, mean (range)	64 (47–83)	62 (47–83)	65 (51–77)	63 (47–82)	63 (49–78)	63 (47–82)
Smoking habits, *n* (%)						
Never	1 (1%)	0 (0%)	1 (3%)	0 (0%)	0 (0%)	0 (0%)
ex	33 (31%)	24 (35%)	9 (24%)	23 (38%)	11 (55%)	12 (30%)
Smoker	72 (68%)	44 (65%)	28 (74%)	37 (62%)	9 (45%)	28 (70%)
Stage of disease, *n* (%)						
I	2 (2%)	1 (1%)	1 (3%)	1 (2%)	0 (0%)	1 (3%)
II	5 (5%)	2 (3%)	3 (8%)	1 (2%)	0 (0%)	1 (3%)
III	55 (53%)	35 (52%)	20 (54%)	31 (53%)	13 (65%)	18 (46%)
IV	42 (40%)	29 (43%)	13 (35%)	26 (44%)	7 (35%)	19 (49%)
Type of lung cancer, *n* (%)						
Small-cell	33 (31%)	23 (34%)	10 (26%)	22 (37%)	11 (55%)	11 (28%)
Adenocarcinoma	33 (31%)	22 (33%)	11 (29%)	18 (31%)	3 (15%)	15 (38%)
Squamous	39 (37%)	22 (33%)	17 (45%)	19 (32%)	6 (30%)	13 (33%)
Small-cell/non-small-cell, *n* (%)						
Small-cell	32 (30%)	22 (32%)	10 (26%)	21 (35%)	10 (50%)	11 (28%)
Non-small-cell	74 (70%)	46 (68%)	28 (74%)	39 (65%)	10 (50%)	29 (72%)

**Table 2:** FACT.

FACT Domains	I	II	III	*p*
PWB, mean (95% CI)	21.8 (20.4–23.2)	19.8 (18.1–21.6)	22.7 (21.3–24.1)	II vs. III *
SWB, mean (95% CI)	25.3 (24.3–26.3)	23.4 (22.7–24.1)	23.4 (22.7–24.1)	I vs. II*, I vs. III *
EWB, mean (95% CI)	18.5 (17.2–19.9)	19.1 (17.9–20.4)	20.5 (19.3–21.7)	I vs. III *
FWB, mean (95% CI)	17.1 (15.2–19.0)	15.6 (13.4–17.9)	18.4 (16.7–20.1)	II vs. III *
LCS, mean (95% CI)	17.0 (15.2–18.8)	19.9 (18.1–21.7)	21.7 (20.6–22.8)	I vs. II *, I vs. III *, II vs. III *
FACT L TOI, mean (95% CI)	55.9 (51.6–60.1)	55.4 (50.4–60.4)	62.8 (59.3–66.4)	I vs. III *, II vs. III *
FACT G, mean (95% CI)	82.7 (78.7–86.7)	78.0 (73.7–82.3)	85.0 (81.6–88.4)	II vs. III*
FACT L TOTAL, mean (95% CI)	99.7 (94.4–104.9)	97.9 (92.2–103.6)	106.7 (102.5–110.9)	I vs. III *, II vs. III *

FACT L—functional assessment of cancer therapy–lung; PWB—physical wellbeing; SWB—social/family wellbeing; EWB—emotional wellbeing; FWB—functional well-being; LCS—lung cancer subscale; FACT L TOI-FACT-L trial outcome index (TOI); FACTG total score–PWB + SWB + EWB + FWB; FACT L TOTAL score–PWB + SWB + EWB + FWB + LCS. I—at diagnosis; II—after cisplatin therapy; III—after TENS therapy. * *p* < 0.05.

**Table 3:** EORTC QLQ-C30.

EORTC QLQ-C30 Domains	I	II	III	*p*
PF, mean (95% CI)	74.7 (67.7–81.7)	74.2 (68.0–80.4)	84.0 (80.0–88.1)	I vs. III *, II vs. III *
RF, mean (95% CI)	67.6 (58.5–76.5)	64.9 (55.2–74.6)	71.1 (62.7–79.4)	II vs. III *
EF, mean (95% CI)	73.9 (65.4–82.4)	76.3 (69.4–83.2)	79.8 (72.8–86.9)	
CF, mean (95% CI)	93.4 (88.6–98.3)	92.1 (86.0–98.2)	94.7 (89.5–100.0)	
SF, mean (95% CI)	82.9 (75.2–90.6)	72.4 (64.2–80.6)	76.8 (70.2–83.3)	
FA, mean (95% CI)	36.5 (26.7–46.4)	33.0 (26.3–39.8)	28.9 (23.3–34.6)	
NV, mean (95% CI)	7.0 (1.1–12.9)	9.2 (2.5–15.9)	6.6 (1.4–11.8)	
PA, mean (95% CI)	25.0 (15.2–34.8)	35.1 (26.8–43.4)	22.8 (18.1–27.5)	II vs. III *
DY, mean (95% CI)	22.8 (11.4–34.2)	12.3 (4.9–19.7)	14.0 (8.6–19.5)	
SL, mean (95% CI)	25.4 (14.5–36.4)	27.2 (17.7–36.7)	15.8 (9.7–21.9)	II vs. III *
AP, mean (95% CI)	24.6 (13.3–35.9)	26.3 (15.5–37.2)	14.0 (7.5–20.6)	II vs. III *
CO, mean (95% CI)	12.3 (4.1–20.5)	9.6 (4.0–15.3)	8.8 (3.9–13.7)	
DI, mean (95% CI)	4.4 (0.6–8.1)	11.4 (5.0–17.8)	4.4 (0.6–8.1)	I vs. II *, II vs. III *
FI, mean (95% CI)	22.8 (12.6–33.0)	35.1 (24.6–45.6)	42.1 (30.8–53.4)	I vs. III *
QL, mean (95% CI)	59.4 (52.8–66.0)	57.0 (53.0–61.1)	62.5 (59.3–65.7)	II vs. III *
QLQ Total, mean (95% CI)	79.6 (73.9–85.3)	78.1 (73.8–82.5)	83.9 (80.9–87.0)	II vs. III *

EORTC QLQ—C30–European Organization for Research and Treatment of Cancer–quality of life questionnaire C30; PF—physical functioning; RF—role functioning; EF—emotional functioning; CF—cognitive functioning; SF—social functioning; FA—fatigue; NV—nausea of vomiting; PA—pain; DY—dyspnea; SL—insomnia; AP—loss of appetite; CO—constipation; DI—diarrhea; FI—financial difficulties; QL—global health status; QLQ Total—QLQ total score. I—at diagnosis; II—after cisplatin therapy; III—after TENS therapy. * *p* < 0.05.

## Data Availability

Not applicable.
